# Supplementary material for: A Class 4-like Chromosomal Integron Found in Aeromonas sp. Genomospecies paramedia Isolated from Human Feces
Source: Microorganisms. 2023 Oct 13;11(10):2548. doi: 10.3390/microorganisms11102548 (PMC10609294; doi:10.3390/microorganisms11102548)
Supplement: Supplementary file 1 [file microorganisms-11-02548-s001.zip › microorganisms-2627378-supplementary.pdf]

Supplementary data

Table S1: Primers used in this study.

	Sequence 5´- 3´	Size (nt)	Tm (°C)	Reference
RT-PCR				
Find_intI4 F	CGCAAGGTATGACAGGTGAC	20	57.7	This study
Find_intI4 R	CTCTGCCTGATGGTCTGGA	19	56.4	
GCAT fw	CTCCTGGAATCCCAAGTATCAG	22	61.6	[48]
GCAT rv	GGCAGGTTGAACAGCAGTATCT	22	64.4	
recA_dw	GGCGTCAAGCACAAGCTGATCG	22	61	
recA_up	TCGAACTCTTGTTCGCTTTCGGC	23	60.1	
Gibson assembly				
Int4_pBAM Fwd	CGAATTCGAGCTCGGTACCCCTGAAACGG ATGTCAGCCTC	40	55.8	This study

Int4_pBAM Rev	GTCGACTCTAGAGGATCCCCGCAAGTTCCG CATAATGCTT	40	54.1	
MLSA				
gyrA2F	ATGAGCGATCTGGCCAGAGA	20	58.2	[8]
gyrA9R	CGCGCCTTGTTCACCTGATA	20	57.7	
gyrB sch3F	CATGTCTACGAGCAGACCTA	20	53.4	
gyrB sch12R	CTCCACGTTCAGGATCTTGCC	21	58	
rpoD70Fs	ACGACTGACCCGGTACGCATGTA	23	62	
rpoD70Rs	ATAGAAATAACCAGACGTAAGTT	23	49.4	
dnaJ-F	CGAGATCAAGAAGGCGTACAAG	22	55.5	
dnaJ-R	CACCACCTTGCACATCAGATC	21	56.1	
dnaX4F	AAAACCCTGGA(AG)GAGCCGCC	20	62.8	
dnaX-11R	AAGGC(GC)AGCAT(GC)CGCAG	17	58.6	

recA3F	GCTGGGTCAGATTGAAAAGC	20	54.4	
recA11R	CTCGCCGTTATAGCTGTACC	20	55	

Gibson assembly: overlapping sequences are underlined.

Table S2. GenBank access number of sequences used in the alignments.

	Gene accesion number															
Strains	gyrA	gyrB	rpoD	dnaJ	dnaX	recA	atpD	dnaK	gltA	groL	metG	ppsA	radA	rpoB	tsf
A. allosaccharophila CECT 4199	NZ_CDBR01000099.1	NZ_CDBR01000042.1	NZ_CDBR01000023.1	NZ_CDBR01000099.1	NZ_CDBR01000042.1	NZ_CDBR01000023.1	HQ442565.1	JN829106.1	JN829300.1	NZ_CDBR01000023.1	NZ_CDBR01000023.1	NZ_CDBR01000023.1	NZ_CDBR01000023.1	NZ_CDBR01000023.1	NZ_CDBR01000023.1
A. aquatica CECT 8025	HG970916.1	HG970927.1	HG970949.1	HG970916.1	HG970927.1	HG970949.1	HG970881.1	------	------	------	------	------	------	------	------
A. aquatilis AE207	LT630730.1	LT630723.1	LT630716.1	LT630730.1	LT630723.1	LT630716.1	LT630758.1	------	------	------	------	------	------	------	------
A. australiensis CECT 8023	NZ_CDDH01000006.1	NZ_CDDH01000074.1	NZ_CDDH01000001.1	NZ_CDDH01000006.1	NZ_CDDH01000074.1	NZ_CDDH01000001.1	NZ_CDDH01000074.1	NZ_CDDH01000074.1	NZ_CDDH01000074.1	NZ_CDDH01000074.1	NZ_CDDH01000074.1	NZ_CDDH01000074.1	NZ_CDDH01000074.1	NZ_CDDH01000074.1	NZ_CDDH01000074.1
A. bestiarum CBA100	NZ_JPWL01000008.1	JPWL01000007.1	NZ_JPWL01000072.1	NZ_JPWL01000008.1	JPWL01000007.1	NZ_JPWL01000072.1	NZ_JPWL01000007.1	NZ_JPWL01000007.1	NZ_JPWL01000007.1	NZ_JPWL01000007.1	NZ_JPWL01000007.1	NZ_JPWL01000007.1	NZ_JPWL01000007.1	NZ_JPWL01000007.1	NZ_JPWL01000007.1
A. bivalvium CECT 7113	NZ_CDBT01000004.1	NZ_CDBT01000055.1	NZ_CDBT01000016.1	NZ_CDBT01000004.1	NZ_CDBT01000055.1	NZ_CDBT01000016.1	NZ_CDBT01000055.1	NZ_CDBT01000055.1	NZ_CDBT01000055.1	NZ_CDBT01000055.1	NZ_CDBT01000055.1	NZ_CDBT01000055.1	NZ_CDBT01000055.1	NZ_CDBT01000055.1	NZ_CDBT01000055.1
A. cavernicola MDC 2508	HQ443164.1	HQ442702.1	HQ442864.1	HQ443164.1	HQ442702.1	HQ442864.1	NZ_PGGC01000100.1	NZ_PGGC01000100.1	NZ_PGGC01000100.1	NZ_PGGC01000100.1	NZ_PGGC01000100.1	NZ_PGGC01000100.1	NZ_PGGC01000100.1	NZ_PGGC01000100.1	NZ_PGGC01000100.1
A. caviae CECT 838	NZ_CDBK01000094.1	NZ_CDBK01000069.1	NZ_CDBK01000071.1	NZ_CDBK01000094.1	NZ_CDBK01000069.1	NZ_CDBK01000071.1	NZ_CDBK01000069.1	NZ_CDBK01000069.1	NZ_CDBK01000069.1	NZ_CDBK01000069.1	NZ_CDBK01000069.1	NZ_CDBK01000069.1	NZ_CDBK01000069.1	NZ_CDBK01000069.1	NZ_CDBK01000069.1
A. crassostreae AOSE3-14A	LT630726.1	LT630719.1	LT630712.1	LT630726.1	LT630719.1	LT630712.1	LT630754.1	-----	-----	-----	-----	-----	-----	-----	-----
A. dhakensis CECT 7289	NZ_CDBP01000029.1	NZ_CDBP01000023.1	NZ_CDBP01000072.1	NZ_CDBP01000029.1	NZ_CDBP01000023.1	NZ_CDBP01000072.1	NZ_CDBP01000023.1	NZ_CDBP01000023.1	NZ_CDBP01000023.1	NZ_CDBP01000023.1	NZ_CDBP01000023.1	NZ_CDBP01000023.1	NZ_CDBP01000023.1	NZ_CDBP01000023.1	NZ_CDBP01000023.1
A. diversa CECT 4254	NZ_CDCE01000034.1	NZ_CDCE01000027.1	NZ_CDCE01000007.1	NZ_CDCE01000034.1	NZ_CDCE01000027.1	NZ_CDCE01000007.1	NZ_CDCE01000027.1	NZ_CDCE01000027.1	NZ_CDCE01000027.1	NZ_CDCE01000027.1	NZ_CDCE01000027.1	NZ_CDCE01000027.1	NZ_CDCE01000027.1	NZ_CDCE01000027.1	NZ_CDCE01000027.1
A. encheleia CECT 4342	NZ_CDDI01000011.1	NZ_CDDI01000004.1	NZ_CDDI01000017.1	NZ_CDDI01000011.1	NZ_CDDI01000004.1	NZ_CDDI01000017.1	NZ_CDDI01000004.1	NZ_CDDI01000004.1	NZ_CDDI01000004.1	NZ_CDDI01000004.1	NZ_CDDI01000004.1	NZ_CDDI01000004.1	NZ_CDDI01000004.1	NZ_CDDI01000004.1	NZ_CDDI01000004.1
A. enterica 113634	LT630725.1	LT630718.1	LT630711.1	LT630725.1	LT630718.1	LT630711.1	LT630753.1	-----	-----	-----	-----	-----	-----	-----	-----
A. enteropelogenes CECT 4487	NZ_CDCG01000018.1	NZ_CDCG01000031.1	NZ_CDCG01000026.1	NZ_CDCG01000018.1	NZ_CDCG01000031.1	NZ_CDCG01000026.1	NZ_CDCG01000010.1	NZ_CDCG01000010.1	NZ_CDCG01000010.1	NZ_CDCG01000010.1	NZ_CDCG01000010.1	NZ_CDCG01000010.1	NZ_CDCG01000010.1	NZ_CDCG01000010.1	NZ_CDCG01000010.1
A. eucrenophila CECT 4224	NZ_CDDF01000001.1	NZ_CDDF01000006.1	NZ_CDDF01000016.1	NZ_CDDF01000001.1	NZ_CDDF01000006.1	NZ_CDDF01000016.1	NZ_CDDF01000006.1	NZ_CDDF01000006.1	NZ_CDDF01000006.1	NZ_CDDF01000006.1	NZ_CDDF01000006.1	NZ_CDDF01000006.1	NZ_CDDF01000006.1	NZ_CDDF01000006.1	NZ_CDDF01000006.1
A. finlandiensis CECT 8028	HG970912.1	HG970923.1	HG970945.1	HG970912.1	HG970923.1	HG970945.1	HG970879.1	-----	-----	-----	-----	-----	-----	-----	-----
A. fluvialis LMG 24681	NZ_CDBO01000034.1	NZ_CDBO01000069.1	NZ_CDBO01000043.1	NZ_CDBO01000034.1	NZ_CDBO01000069.1	NZ_CDBO01000043.1	NZ_CDBO01000069.1	NZ_CDBO01000069.1	NZ_CDBO01000069.1	NZ_CDBO01000069.1	NZ_CDBO01000069.1	NZ_CDBO01000069.1	NZ_CDBO01000069.1	NZ_CDBO01000069.1	NZ_CDBO01000069.1
A. hydrophila CECT 839	HQ443174.1	JN711776.1	JN215537.1	HQ443174.1	JN711776.1	JN215537.1	HQ442559.1	-----	-----	-----	-----	-----	-----	-----	-----
A. intestinalis 1178C	LT630724.1	LT630717.1	LT630710.1	LT630724.1	LT630717.1	LT630710.1	LT630752.1	-----	-----	-----	-----	-----	-----	-----	-----
A. jandaei CECT 4228	NZ_CDBV01000025.1	NZ_CDBV01000026.1	AY169341.1	NZ_CDBV01000025.1	NZ_CDBV01000026.1	AY169341.1	NZ_CDBV01000026.1	NZ_CDBV01000026.1	NZ_CDBV01000026.1	NZ_CDBV01000026.1	NZ_CDBV01000026.1	NZ_CDBV01000026.1	NZ_CDBV01000026.1	NZ_CDBV01000026.1	NZ_CDBV01000026.1
A. lacus CECT 8024	HG970914.1	HG970925.1	HG970947.1	HG970914.1	HG970925.1	HG970947.1	HG970883.1	-----	-----	-----	-----	-----	-----	-----	-----
A. lusitana MDC 2473							NZ_PGCP01000001.1	NZ_PGCP01000001.1	NZ_PGCP01000001.1	NZ_PGCP01000001.1	NZ_PGCP01000001.1	NZ_PGCP01000001.1	NZ_PGCP01000001.1	NZ_PGCP01000001.1	NZ_PGCP01000001.1
A. media AK208	KP401432.1	KP400939.1	KP401011.1	KP401489.1	KP401068.1	KP400849.1	KP401125.1	KP401250.1	KP401250.1	KP401360.1	KP401288.1	KP400777.1	KP401544.1	KP401180.1	KP400904.1
A. media CR4.2-17C	KP401444.1	KP400950.1	KP401023.1	KP401501.1	KP401080.1	KP400861.1	KP401137.1	KP401261.1	KP401261.1	KP401372.1	KP401300.1	KP400789.1	KP401555.1	KP401191.1	KP400915.1
A. media 1086C	KP401428.1	KP400936.1	KP401007.1	KP401485.1	KP401064.1	KP400845.1	KP401121.1	KP401247.1	KP401247.1	KP401356.1	KP401284.1	KP400773.1	KP401541.1	KP401177.1	KP400901.1
A. media 4232	NZ_CDBZ01000214.5	NZ_CDBZ01000214.4	NZ_CDBZ01000214.3	NZ_CDBZ01000214.2	NZ_CDBZ01000214.1	NZ_CDBZ01000214.0	NZ_CDBZ01000214.1	NZ_CDBZ01000214.1	NZ_CDBZ01000214.1	NZ_CDBZ01000214.1	NZ_CDBZ01000214.1	NZ_CDBZ01000214.1	NZ_CDBZ01000214.1	NZ_CDBZ01000214.1	NZ_CDBZ01000214.1
A. media CECT 4234	KP401452.1	AY101824.1	AY249191.1	KP401452.1	AY101824.1	AY249191.1	KP401145.1	KP401269.1	KP401269.1	KP401380.1	KP401308.1	KP400797.1	KP401563.1	KP401199.1	KP400923.1
A. media UTS15	KP401441.1	KP400947.1	KP401020.1	KP401498.1	KP401077.1	KP400858.1	KP401134.1	KP401258.1	KP401258.1	KP401369.1	KP401297.1	KP400786.1	KP401552.1	KP401188.1	KP400912.1
A. molluscorum CIP 108676	-----	-----	-----	-----	-----	-----	-----	-----	-----	KP401394.1	KP401322.1	KP400811.1	KP401572.1	-----	-----
A. piscicola LMG 24783	NZ_CDBL01000018.1	NZ_CDBL01000090.1	NZ_CDBL01000080.1	NZ_CDBL01000018.1	NZ_CDBL01000090.1	NZ_CDBL01000080.1	NZ_CDBL01000090.1	NZ_CDBL01000090.1	NZ_CDBL01000090.1	NZ_CDBL01000090.1	NZ_CDBL01000090.1	NZ_CDBL01000090.1	NZ_CDBL01000090.1	NZ_CDBL01000090.1	NZ_CDBL01000090.1
A. popoffii CIP 105493	NZ_CDBI01000024.1	NZ_CDBI01000062.1	NZ_CDBI01000001.1	NZ_CDBI01000024.1	NZ_CDBI01000062.1	NZ_CDBI01000001.1	NZ_CDBI01000062.1	NZ_CDBI01000062.1	NZ_CDBI01000062.1	NZ_CDBI01000062.1	NZ_CDBI01000062.1	NZ_CDBI01000062.1	NZ_CDBI01000062.1	NZ_CDBI01000062.1	NZ_CDBI01000062.1
A. rivipollensis KN-Mc-11N1	NZ_CP027856.1	NZ_CP027856.1	NZ_CP027856.1	NZ_CP027856.1	NZ_CP027856.1	NZ_CP027856.1	NZ_CP027856.1	NZ_CP027856.1	NZ_CP027856.1	NZ_CP027856.1	NZ_CP027856.1	NZ_CP027856.1	NZ_CP027856.1	NZ_CP027856.1	NZ_CP027856.1
A. *rivuli* DSM 22539	NZ_CDBJ01000054.1	NZ_CDBJ01000087.1	NZ_CDBJ01000065.1	NZ_CDBJ01000054.1	NZ_CDBJ01000087.1	NZ_CDBJ01000065.1	NZ_CDBJ01000087.1	NZ_CDBJ01000087.1	NZ_CDBJ01000087.1	NZ_CDBJ01000087.1	NZ_CDBJ01000087.1	NZ_CDBJ01000087.1	NZ_CDBJ01000087.1	NZ_CDBJ01000087.1	NZ_CDBJ01000087.1
A. *salmonicida* CIP 103209	NZ_CDDW01000106.1	NZ_CDDW01000008.1	NZ_CDDW01000026.1	NZ_CDDW01000106.1	NZ_CDDW01000008.1	NZ_CDDW01000026.1	NZ_CDDW01000008.1	NZ_CDDW01000008.1	NZ_CDDW01000008.1	NZ_CDDW01000008.1	NZ_CDDW01000008.1	NZ_CDDW01000008.1	NZ_CDDW01000008.1	NZ_CDDW01000008.1	NZ_CDDW01000008.1
A. *sanrellii* LMG 24682	NZ_CDBN01000004.1	NZ_CDBN01000092.1	NZ_CDBN01000049.1	NZ_CDBN01000004.1	NZ_CDBN01000092.1	NZ_CDBN01000049.1	NZ_CDBN01000092.1	NZ_CDBN01000092.1	NZ_CDBN01000092.1	NZ_CDBN01000092.1	NZ_CDBN01000092.1	NZ_CDBN01000092.1	NZ_CDBN01000092.1	NZ_CDBN01000092.1	NZ_CDBN01000092.1
A. *schubertii* CECT 4240	NZ_CDDB01000010.1	NZ_CDDB01000021.1	NZ_CDDB01000081.1	NZ_CDDB01000010.1	NZ_CDDB01000021.1	NZ_CDDB01000081.1	NZ_CDDB01000021.1	NZ_CDDB01000021.1	NZ_CDDB01000021.1	NZ_CDDB01000021.1	NZ_CDDB01000021.1	NZ_CDDB01000021.1	NZ_CDDB01000021.1	NZ_CDDB01000021.1	NZ_CDDB01000021.1
A. *simiae* CIP 107798	NZ_CDBY01000023.1	NZ_CDBY01000061.1	NZ_CDBY01000062.1	NZ_CDBY01000023.1	NZ_CDBY01000061.1	NZ_CDBY01000062.1	NZ_CDBY01000061.1	NZ_CDBY01000061.1	NZ_CDBY01000061.1	NZ_CDBY01000061.1	NZ_CDBY01000061.1	NZ_CDBY01000061.1	NZ_CDBY01000061.1	NZ_CDBY01000061.1	NZ_CDBY01000061.1
A. *sobria* CECT 4245	NZ_CDBW01000041.1	HQ442698.1	NZ_CDBW01000045.1	NZ_CDBW01000041.1	NZ_CDBW01000036.1	NZ_CDBW01000045.1	HQ442569.1	JN829127.1	JN829127.1	------	NZ_CDBW01000041.1	-------	JN829711.1	JN829903.1	JN830098.1
A. *taiwanensis* LMG 24683	NZ_BAWK01000039.1	NZ_BAWK01000086.1	NZ_BAWK01000094.1	NZ_BAWK01000039.1	NZ_BAWK01000086.1	NZ_BAWK01000094.1	NZ_BAWK01000086.1	NZ_BAWK01000086.1	NZ_BAWK01000086.1	NZ_BAWK01000086.1	NZ_BAWK01000086.1	NZ_BAWK01000086.1	NZ_BAWK01000086.1	NZ_BAWK01000086.1	NZ_BAWK01000086.1
A. *tecta* CECT 7082	NZ_CDCA01000010.1	NZ_CDCA01000023.1	NZ_CDCA01000028.1	NZ_CDCA01000010.1	NZ_CDCA01000023.1	NZ_CDCA01000028.1	NZ_CDCA01000023.1	NZ_CDCA01000023.1	NZ_CDCA01000023.1	NZ_CDCA01000023.1	NZ_CDCA01000023.1	NZ_CDCA01000023.1	NZ_CDCA01000023.1	NZ_CDCA01000023.1	NZ_CDCA01000023.1
A. *veronii* CECT 4257	HQ443160.1	AY101795.1	HQ442833.1	HQ443160.1	AY101795.1	HQ442833.1	HQ442580.1	-----	-----	-----	-----	-----	-----	-----	-----
Oceanimonas sp. GK1	NC_016745.1	NC_016745.1	NC_016745.1	NC_016745.1	NC_016745.1	NC_016745.1	NC_016745.1	NC_016745.1	NC_016745.1	NC_016745.1	NC_016745.1	NC_016745.1	NC_016745.1	NC_016745.1	NC_016745.1

Table S3. Diversity of cassette promoters in different integrons.

The polymorphisms in the promoters are indicated in gray. Promoter sequence

Promoter	-35	Spacer	-10	Position
Consensus	TTGACA	17(+_1)	TATAAT	
Pc4L-1	TTGCCA	16	CAACAT	intI1
Pc4L-2	ATGAGT	18	TATAAA	intI1-attI
Pc4L-3	ATGAAG	18	CATAAT	attI
Pc4L-4	TTTCAA	15	TCTCAC	attI
P2	TTGTTA		TACAGT	attI1
PcH2	TTGACA	17	TAAGCT	intI1
PcH1	TGGACA	17	TAAACT	intI1
PcSS	TTGATA	17	TAAACT	intI1
PcPUO	TCGACA	17	TAAACT	intI1
PcW	TGGACA	17	TAAGCT	intI1
PcIn42	TTGGCA	17	TAAACT	intI1
PcIn116	TTGACA	17	TGAACT	intI1
Pc2	TTTTAA	17	TAAAAT	attI2
Pc3	TAGACA	17	TAGGCT	intI3
Pc intIA	TTGAGC	16	TCTGAT	attI

were consulted in reference [68].

Table S4. Ribosome binding sites of cassettes.

Cassette/gene	Sequence<br><br>---AGGAGG--------ATG
intI4-like	ACATGTGGAGGTGACTCATG
orf1	TTAGAGGGTTTATATTTATG
orf2	CAAGGAAGACAATCATTATG
lpt	CATATGGAGAAATTAGTATG
aadA1	GCAAGGCGTTAAACATCATG
orf5	AGCGTTAGAGTGCAAATATG
orf 6	AGCCTCCACGGACAGCCATG
orf 7	AATTACGCGAGGACTGAATG
orf 8	CATAGAAGGGGGCAAAAATG
orf 9	GATTCAAGAGTGATTTTATG
orf 10	CTGCACAGGTGAATGGCATG

Ribosome binding sites are underlined.

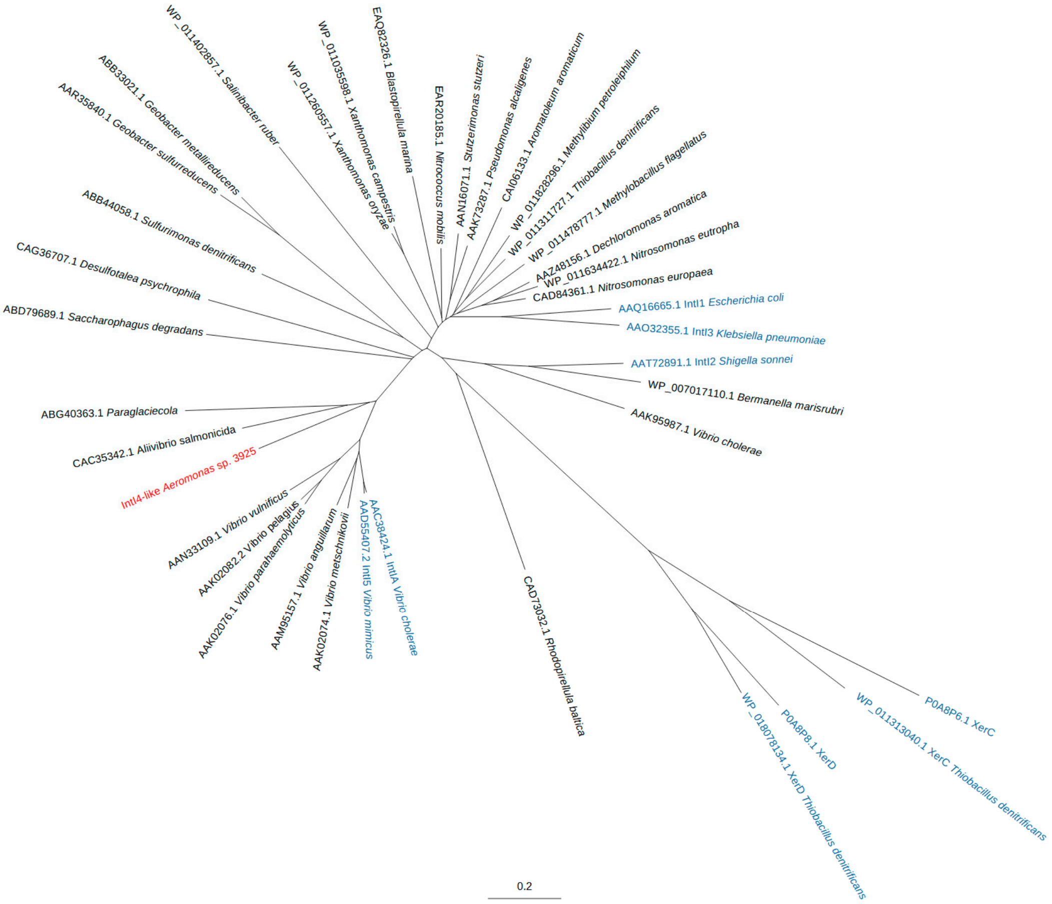

Figure S1. Phylogenetic relationships among IntI integrase. This tree was constructed using amino acid sequences recovered from annotated as integron integrases (black) at NCBI protein database and reference sequences (blue) (IntI1, IntI2, IntI3, IntI4, IntI5, and XerD sequences).
